# Understanding the value of curation: A survey of researcher perspectives of data curation services from six US institutions

**DOI:** 10.1371/journal.pone.0293534

**Published:** 2023-11-01

**Authors:** Wanda Marsolek, Sarah J. Wright, Hoa Luong, Susan M. Braxton, Jake Carlson, Sophia Lafferty-Hess

**Affiliations:** 1 Univeristy of Minnesota Libraries, University of Minnesota, Minneapolis, MN, United States of America; 2 Cornell University Library, Cornell University, Ithaca, NY, United States of America; 3 University Library, University of Illinois–Urbana Champaign, Urbana, IL, United States of America; 4 University at Buffalo Libraries, University at Buffalo, Buffalo, NY, United States of America; 5 Duke University Libraries, Duke University, Durham, NC, United States of America; Boyce Thompson Institute, UNITED STATES

## Abstract

Data curation encompasses a range of actions undertaken to ensure that research data are fit for purpose and available for discovery and reuse, and can help to improve the likelihood that data is more FAIR (Findable, Accessible, Interoperable, and Reusable). The Data Curation Network (DCN) has taken a collaborative approach to data curation, sharing curation expertise across a network of partner institutions and data repositories, and enabling those member institutions to provide expert curation for a wide variety of data types and discipline-specific datasets. This study sought to assess the satisfaction of researchers who had received data curation services, and to learn more about what curation actions were most valued by researchers. By surveying researchers who had deposited data into one of six academic generalist data repositories between 2019–2021, this study set out to collect feedback on the value of curation from the researchers themselves. A total of 568 researchers were surveyed; 42% (238) responded. Respondents were positive in their evaluation of the importance and value of curation, indicating that the participants not only value curation services, but are largely satisfied with the services provided. An overwhelming majority 97% of researchers agreed that data curation adds value to the data sharing process, 96% agreed it was worth the effort, and 90% felt more confident sharing their data due to the curation process. We share these results to provide insights into researchers’ perceptions and experience of data curation, and to contribute evidence of the positive impact of curation on repository depositors. From the perspective of researchers we surveyed, curation is worth the effort, increases their comfort with data sharing, and makes data more findable, accessible, interoperable, and reusable.

## Introduction

The Data Curation Network’s (DCN) collaborative approach to create a scalable solution for domain specific, expert data curation shared across a network of partner institutions and data repositories ensures that a wide variety of data types and discipline-specific datasets can be curated by experts who would not otherwise be available across individual institutions [1]. In order to evaluate the satisfaction level of researchers who had received data curation services, 568 researchers were surveyed who deposited data into one of six academic generalist data repositories over the past two years. By surveying researchers who had recent experience with data curation services, we hoped to explore not only their satisfaction level, but also their attitudes and perceptions towards data curation. For the purpose of the survey, a definition of data curation was provided: “the various actions taken to ensure that data are fit for purpose and available for discovery and reuse”. The 11-question survey received an overall response rate of 42% with 239 responses. Overwhelmingly, respondents were positive, even glowing, in their evaluation of the importance and value of curation, indicating that the participants not only value curation services, but are largely satisfied with curation services provided by the six libraries and institutional repositories in collaboration with the Data Curation Network [1].

There is anecdotal evidence that data curation is an important step towards making data FAIR (Findable, Accessible, Interoperable, and Reusable) [2]. However, there is a lack of literature about researcher satisfaction with data curation services. The following investigates researcher experiences with curation services in generalist data repositories.

## Literature review

### What is curation? Why is it important?

Johnston et al. defines data curation as “the encompassing work and actions taken by curators of a data repository in order to provide meaningful and enduring access to data” [3]. This is one of many definitions of data curation, but most agree that curation is a set of actions to add value to data. The DCN website defines data curation as actions that “enable data discovery and retrieval, maintain data quality, add value, and provide for re-use over time through activities including authentication, archiving, metadata creation, digital preservation, and transformation” [1]. While the members of the DCN have agreed upon a definition of data curation and follow the DCN’s CURATED model [4] for curating data, institutions often have their own workflows. Curation steps may occur in a different order or at different times during the publication process depending on the particular dataset or needs of the researcher. For instance, Illinois encourages researchers to request a pre-publication review to catch any initial issues but also performs curation when depositors have opted to publish their dataset prior to review [5]. The Data Repository for the University of Minnesota (DRUM) appraises all submitted datasets prior to them being accepted including verification of no disclosure risk, sensitive data, or copyright concerns. Curation begins once the datasets are accepted and public. In contrast, at this point in time Cornell’s institutional repository, eCommons, does not require curation for deposit into the repository, although it is highly recommended. Despite these differences, curation plays a central role, adding value by making data accessible, discoverable, reusable, and understandable which increases the data’s FAIRness. In a mixed methods study, Melero & Navarro‐Molina looked at data sharing and reuse in the food science & technology field and found that the majority of respondents believed that it is labor intensive to make their data reusable by others [6]. Hemphil et al. concluded that the more curation work done on a dataset such as adding metadata and providing more context on how the data was collected, the more the data was reused [7]. In a focus group study led by Johnston et al. in 2016, they found that while those researchers felt that curation was important, only 18% of the researchers were satisfied with the curation services they received and identified several important data curation activities that libraries could develop or better advertise [3].

### Why do researchers share their data (or not)?

While the literature is limited regarding researchers satisfaction with data curation, there is an abundance of literature that provides plenty of reasons for sharing data as a way to advance research and knowledge, dissemination, collaboration, accessibility, transparency, and recognition [8–10]. In addition, there are also funder mandates [11,12] and journal requirements [13–15]. Tenopir et al. consider the most important aspects of sharing data is the overall access, reuse, and preservation of data [9].

Researchers, when asked, suggest they are supportive of data sharing and data reuse; however, when examining the numbers of various surveys and studies, fewer are actually sharing [8,16]. The reasons researchers do not share their data are many, including lack of: training, time, standards, incentive, and money; fear of: getting scooped, revelation of error, misuse or misrepresentation of data; privacy; and overall effort [8,9,17]. Fecher et al. suggest that a core reason for researchers’ data sharing hesitancy is the need to be in control of access and use [16] of their data. Other reasons researchers attribute to not sharing their data are lack of interoperability of the data and lack of metadata standards known to them [16]. Many of these impediments could be mitigated by working with a curator who offers recommendations to improve the dataset before its publication. As Mullendore et al. note, “Curation tasks should involve partnerships between scientific and data experts to take advantage of their respective knowledge and skills” [18].

### What value does curation add to the data sharing process?

Several studies have discussed the benefits of curation through the lens of repositories, librarians, and ease of future reuse. Results of a study by Boyd who examined over 29,000 datasets in the Harvard Dataverse Repository affirmed that experienced data curators are valuable to both repositories and to the current and future research community [19]. Data curation adds value to data [5,17,20] through several touch points consisting of but not limited to peer review, metadata inspection and enhancement, documentation enrichment, preservation, and discoverability. Trisovic et al., found that data repositories and services (i.e. curators) “contribute significantly to the quality, longevity, and reusability of datasets” [20]. By performing curatorial checks, curators identify issues found during data submissions, such as missing documentation, missing files/attributes/values, typos, and need for additional metadata to enhance FAIRness of the dataset.

Another important lens to consider when evaluating the benefits of (and need for) curation, is that of the researcher. When submitting their datasets to repositories researchers have found that curators can help improve the quality of the shared data [10,20]. Specific actions such as enhancing both discoverability and dissemination, heightening accessibility and preservation, and augmenting documentation and metadata [10] all improve quality. According to Trisovic et al. datasets that had been reviewed included more keywords, more versions, and more metadata than those that had not [20]. The extra steps that curators take can make the dataset more FAIR and of higher quality. Kratz and Strasser surveyed 250 researchers to gain insight into what researchers’ expectations are for publishing/sharing their data. They found that researchers expect the data to be available, documentation reviewed, and metadata enhanced [17]. Published data helps with proper citation when used by others as well as download count; both attributes help show the impact of the data. Kratz and Strasser found from their survey that peer review of data by curators “establishes the trustworthiness of datasets and elevates its perceived value more than any other factor” [17]. This is facilitated by providing succinct, prioritized, and “actionable suggestions without overwhelming busy researchers” [5]. Like peer review, data curation offers the researcher an opportunity to view their output through another set of eyes. Repositories and researchers have found that curation does improve datasets [5].

### What barriers do researchers experience in curating their own data before sharing?

Researchers wrestle with documentation and metadata in their own work due to lack of access to standards and templates, which is a drain on resources like time and labor [4,21]. Mullendore et al. reported that some researchers spend significant amounts of their time curating their data, sometimes “over 50% of their funded time” [18]. When Johnston et al. asked researchers what curators could do to help them in their work, overwhelmingly they responded with anything they could hand off to a curator so the researcher didn’t have to deal with it [3].

The process of preparing data for sharing is not simple, and works best when it is planned for at the outset of a project, and considered throughout the research lifecycle. More often than not, researchers report their findings by publishing an article before publicly sharing their data [9,10]. Researchers may not share their data due to an overall failure to include sufficient documentation and metadata while collecting and analyzing data [9]. There is a tendency not to value this additional labor as it is not typically recognized in tenure and promotion of researchers [21]. Once an article is published, researchers move on and the previous work is out of sight, out of mind. Thus, keeping up with documentation *during* the research process is vital for the researcher creating the data and the future users using the data and documentation [22]. Otherwise, nuances that may help future users, including themselves, understand the data may be lost. When submitting data to repositories, researchers are less likely to provide suitable documentation unless they are required to [20,23]. While academic journal standards still vary, some require reproducibility verification, where researchers must provide their materials (e.g. readme files, supporting data, code, or software) so that it can be verified and reproduced before being published [13].

The authors wanted to better understand the value of curation from the researchers’ perspective. Clearly there is some evidence that curation is valuable, but not enough studies explore researcher attitudes towards curation services. The multi-institutional structure of the DCN offers a unique opportunity to examine researcher perspectives at multiple institutions using the same survey design and methodology on a larger population. Understanding the perceptions of researchers who utilize curation services can help mitigate existing barriers, provide better user centered services, and possibly make a case for more widespread adoption of curation services.

## Methodology

To assess the user’s experience including their perspective regarding data sharing, their attitudes toward data curation, and the level of satisfaction with the data curation service provided at each of the authors’ institutions, the survey instrument was drafted consisting of multiple choice and open-ended questions. The results of this survey were intended to evaluate and improve data repository service; the IRB determined this research does not involve human subjects as defined by DHHS and FDA regulations. The survey was implemented in Qualtrics [24] and distributed to data depositors at six Data Curation Network institutions, including: Cornell University’s eCommons; Duke University’s Research Data Repository; Johns Hopkins University’s Data Archive; University of Illinois Urbana-Champaign’s Illinois Data Bank, University of Michigan’s Deep Blue Data, and the University of Minnesota’s Data Repository for U of M (DRUM).

Researchers who deposited a dataset within the repositories at the above institutions between January 1, 2019 and March 15, 2021 were targeted. This time range was selected to strike a balance between distributing to the largest number of depositors and surveying depositors whose recollection of their experience was still relatively fresh. The authors also hypothesized that researchers would be more likely to participate if the survey invitation came from their institution, rather than from the DCN as a whole. Therefore, each institution was responsible for distributing the survey to their depositors. Surveys were kept open for ~2 weeks between April-June 2021. Each survey participant response was linked to a submitted dataset with a DOI for the purposes of future analysis and identification. A set of primary questions was used by all institutions. Raw data from each institution was combined into a single spreadsheet, standardized in SPSS, and analyzed using Excel. In checking for internal consistency, one outlier was discovered where it seemed that the respondent flipped the orientation of the Likert scale such that their free text response strongly contradicted their other responses; given this ambiguity, this response was removed to result in 238 valid responses for the survey. Not all participants answered all questions, and null responses to individual questions were excluded from analyses, so total responses vary by question. A team of three reviewed all the free text responses, manually characterizing them into specific themes/subthemes, and using a two-stage process (independent followed by group review) to ensure consistency of application of the themes across all responses. As part of the qualitative coding, the authors applied the actions identified in the Value of Curation survey [25] and made small adjustments through an iterative design model (see S1 Appendix for full definitions that were used during the qualitative analysis).

## Results

### Distribution and response rate

A total of 568 emails were sent to researchers across six academic data repositories, with a robust response rate of 42% with 238 responses. Table 1 displays the distribution count and response rate for the DCN’s “Data Curation Service Survey” by repository.

**Table 1:** Distribution count and response rate for the data curation network’s “data curation service survey”.

Repository Affiliation	Date Sent	Distribution Count	Response Count	Response Rate
University of Minnesota Data Repository for the U of M (DRUM)	2021-04-26	197	81	42%
University of Michigan Deep Blue Data	2021-05-11	130	63	48%
University of Illinois at Urbana-Champaign (UIUC) Illinois Data Bank	2021-05-18	121	45	37%
Johns Hopkins University Data Archive	2021-06-17	32	12	38%
Duke University Research Data Repository	2021-05-05	54	19	35%
Cornell University eCommons	2021-04-26	34	18	53%
**Total**		**568**	**238**	**42%**

### Did you expect local repository staff to curate your data?

**Fig 1:**
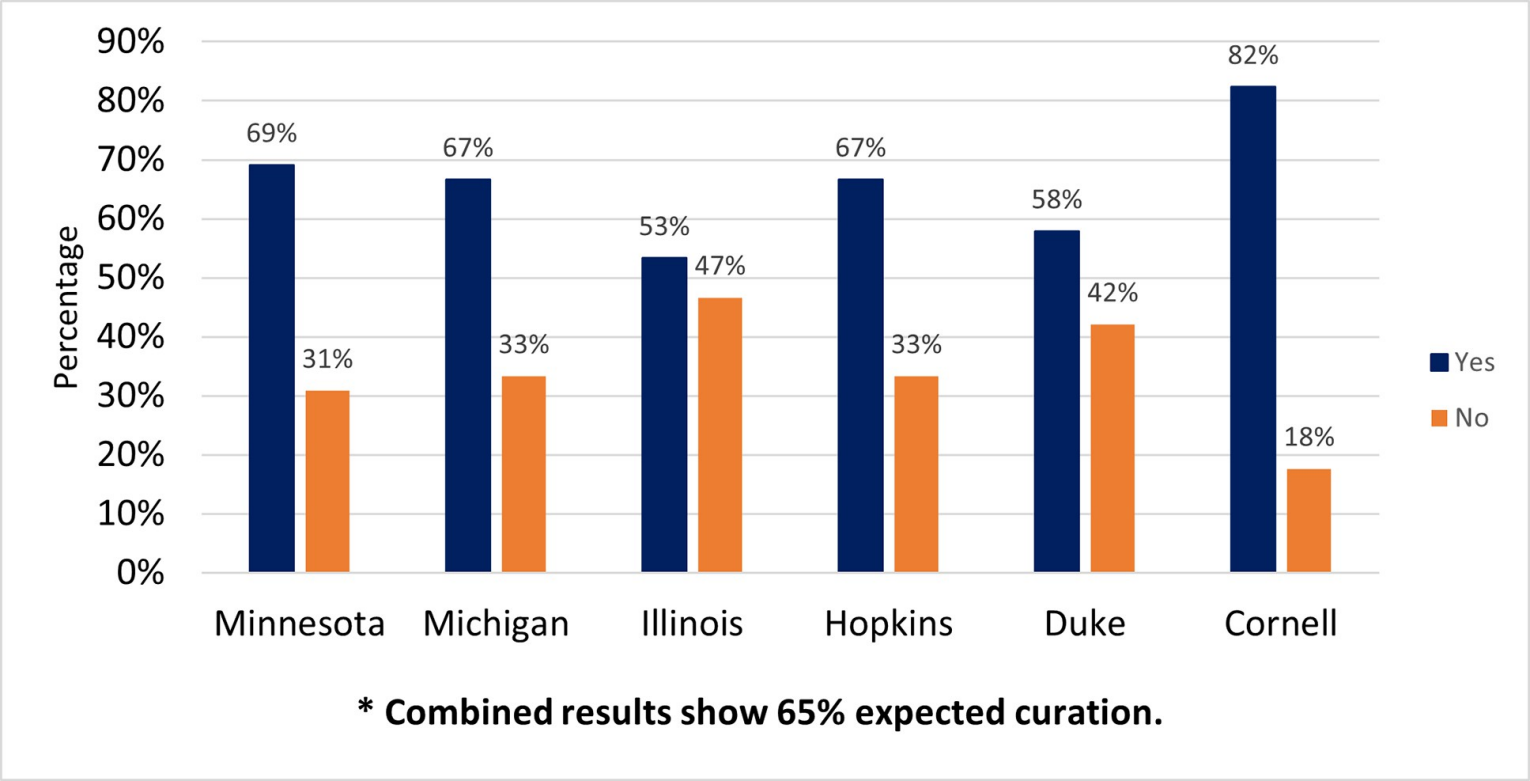
Did you expect [repository name] staff to curate your dataset "[title of dataset]"?. Yes/No.

Data curation is defined differently depending on the discipline. To ensure a common understanding, the authors collectively defined the term “data curation” based on their experience at the beginning of the survey as “the various actions taken to ensure that data are fit for purpose and available for discovery and reuse. During the curatorial review process, a data curator may check files, review metadata, and/or make suggestions that would help others find and reuse your data.” After ensuring a common starting place, the first question asked if the participants expected their local repository staff to curate their dataset. More than half of the participants (65%, n = 156) expected curation, while 35% (n = 82) did not. Fig 1 displays participants’ expectations for curation broken down by institution.

### I was satisfied with the curatorial review my data received

**Fig 2:**
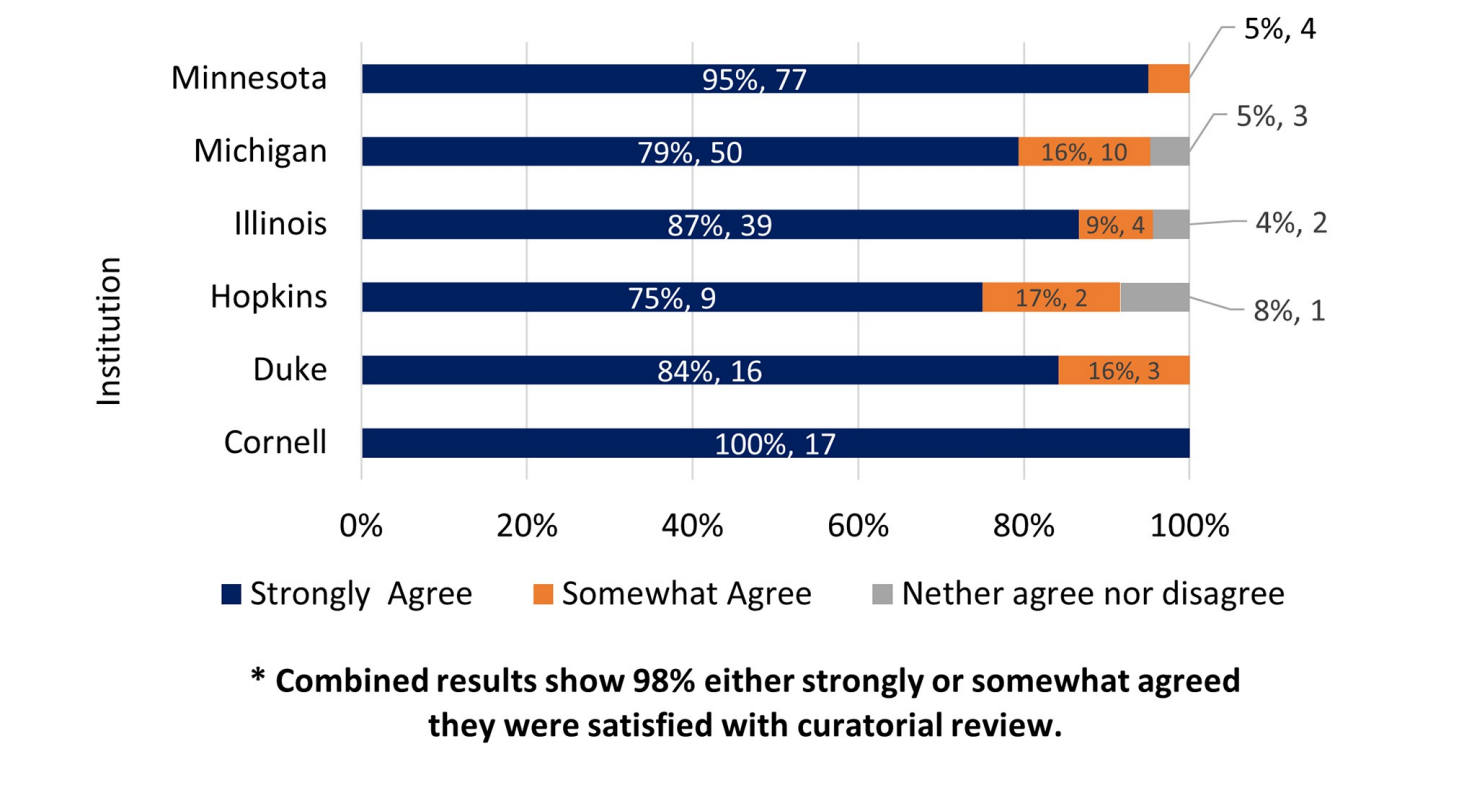
I was satisfied with the curatorial review my data received. Strongly or somewhat agree; neither agree nor disagree; and strongly or somewhat disagree (zero respondents selected). Graph is displayed based on institutional responses.

When asked how satisfied participants were with the curatorial review, a majority of participants (98%, n = 231) either strongly (88%) or somewhat (10%) agreed that they were satisfied with the review that their dataset received, while only 3% (n = 6) were neutral (based on 237 valid responses to the question). No participants indicated that they strongly or somewhat disagreed that they were satisfied. Fig 2 displays the breakdown for each institution based on the satisfaction level.

### Were any changes made to your data submission due to the curatorial review?

**Fig 3:**
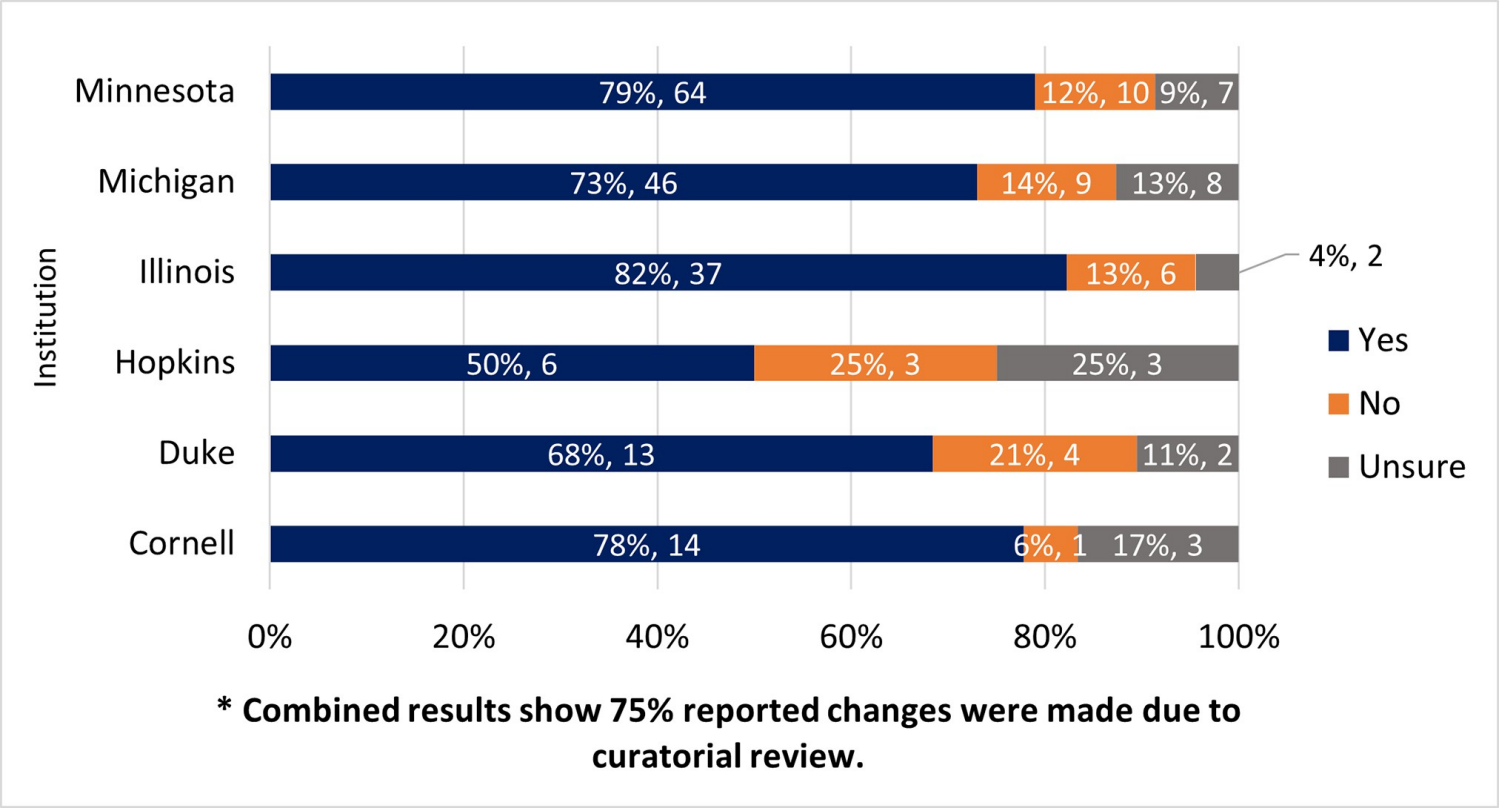
Were any changes made to the data submissions due to the curatorial review?. Yes, no, and unsure. The graph is displayed based on institutional responses.

The next question asked whether any changes were made to the dataset as a result of curation. Skip logic was applied to follow up on the type of changes, and, if no changes were made, why. 75% (n = 180) participants across six institutions reported that changes had been made to their dataset as the result of the curatorial review, while 14% (n = 34) mentioned no changes were made and 10% (n = 25) were unsure. Fig 3 shows that at each institution, at least 50% of the time, changes were made, either by the data depositors themselves or curators, due to the curatorial review.

### If yes, what changes were made?

**Fig 4:**
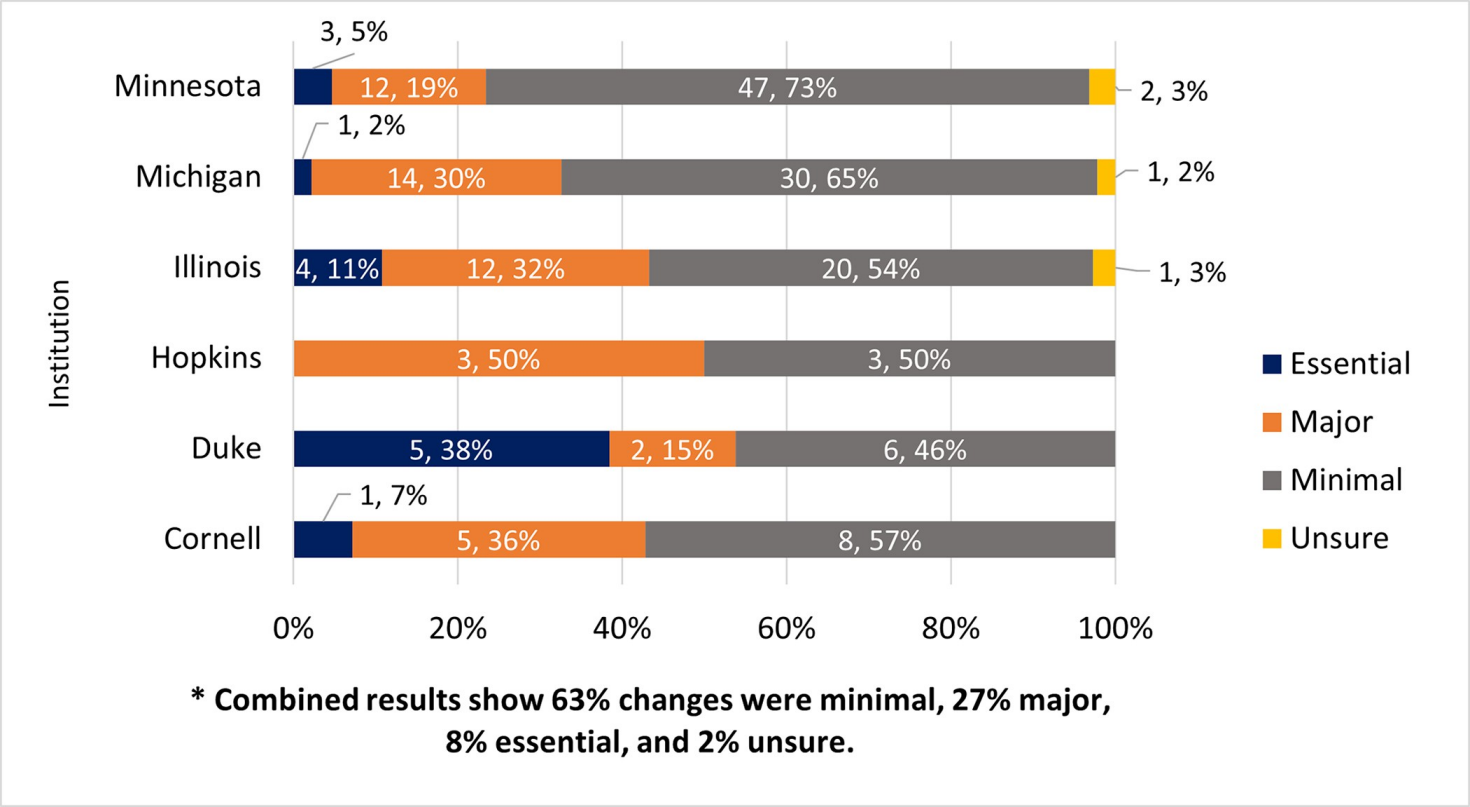
What changes were made due to the curatorial review?. Essential changes (e.g., an error was corrected); major changes (e.g., files updated/added), minimal changes (e.g., small edits/additions); and unsure. Within-institution results are shown.

The 180 participants who reported that changes were made to their dataset were then asked to categorize their changes according to predefined categories (Fig 4). Those categories included essential changes (e.g., an error was corrected), major changes (e.g., files updated/added), minimal changes (e.g., small edits/additions), and unsure (indicating that they were not sure what changes had been made). Minimal changes were reported by 63% (n = 114), followed by major changes (27%, n = 48), essential changes (8%, n = 14), and four (2%) indicated that they were not sure about the changes. Changes made due to curatorial review can vary greatly: from correction of typos to the addition or removal of files, to creation of metadata and documentation necessary to understand and reuse the data, and everything in between.

### If no changes were made, why not?

For the 34 participants who did not make changes to their dataset after the curation, a list of common reasons was provided (No changes were needed; I did not agree with the recommended changes; I did not have time to work on this; Unsure; Other + free text option). Of the participants that made no changes to their dataset, 94% (n = 32) simply indicated that no changes were needed for their dataset. One participant responded that they didn’t have time to work on making the changes. Another participant stated “*Project team member sits on data curation team and provided expert knowledge*,” which was interpreted as no changes were needed.

### Due to the curation process I felt more confident sharing my data

**Fig 5:**
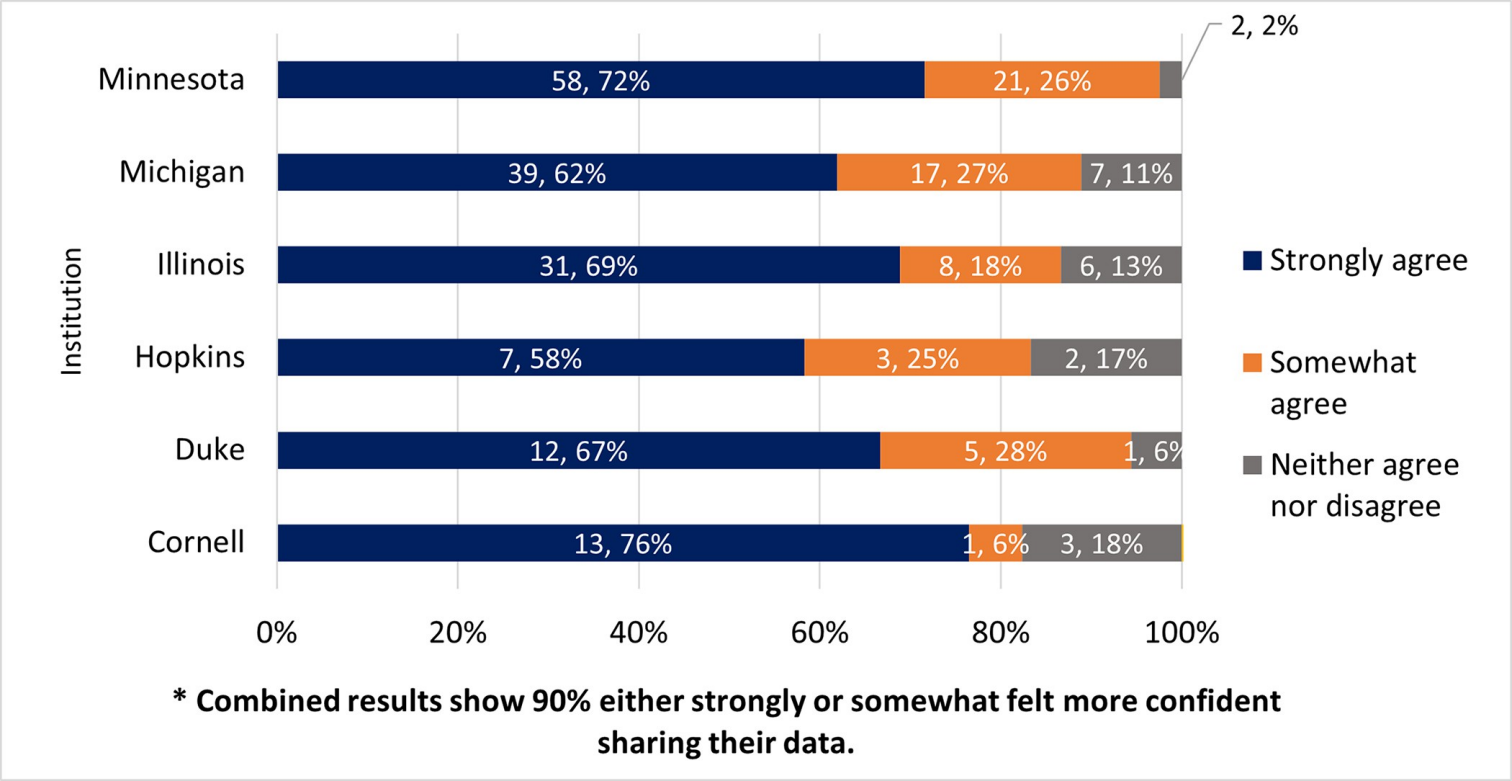
Due to the curation process, the participants felt more confident sharing their data?. Strongly or somewhat agree; neither agree nor disagree; and strongly or somewhat disagree (zero respondents selected). The graph is displayed based on institutional responses.

The next question concerned participant’s level of confidence when sharing their curated dataset. There were 237 responses to this question. A large majority of participants (90%, n = 215) either strongly (67%, n = 160) or somewhat (23%, n = 55) agreed that they felt more confident sharing their data due to the curation process; 9% (n = 21) neither agreed nor disagreed and none strongly or somewhat agreed. Fig 5 displays the breakdown based on each institution’s responses.

### Data curation by this repository adds value to the data sharing process

**Fig 6:**
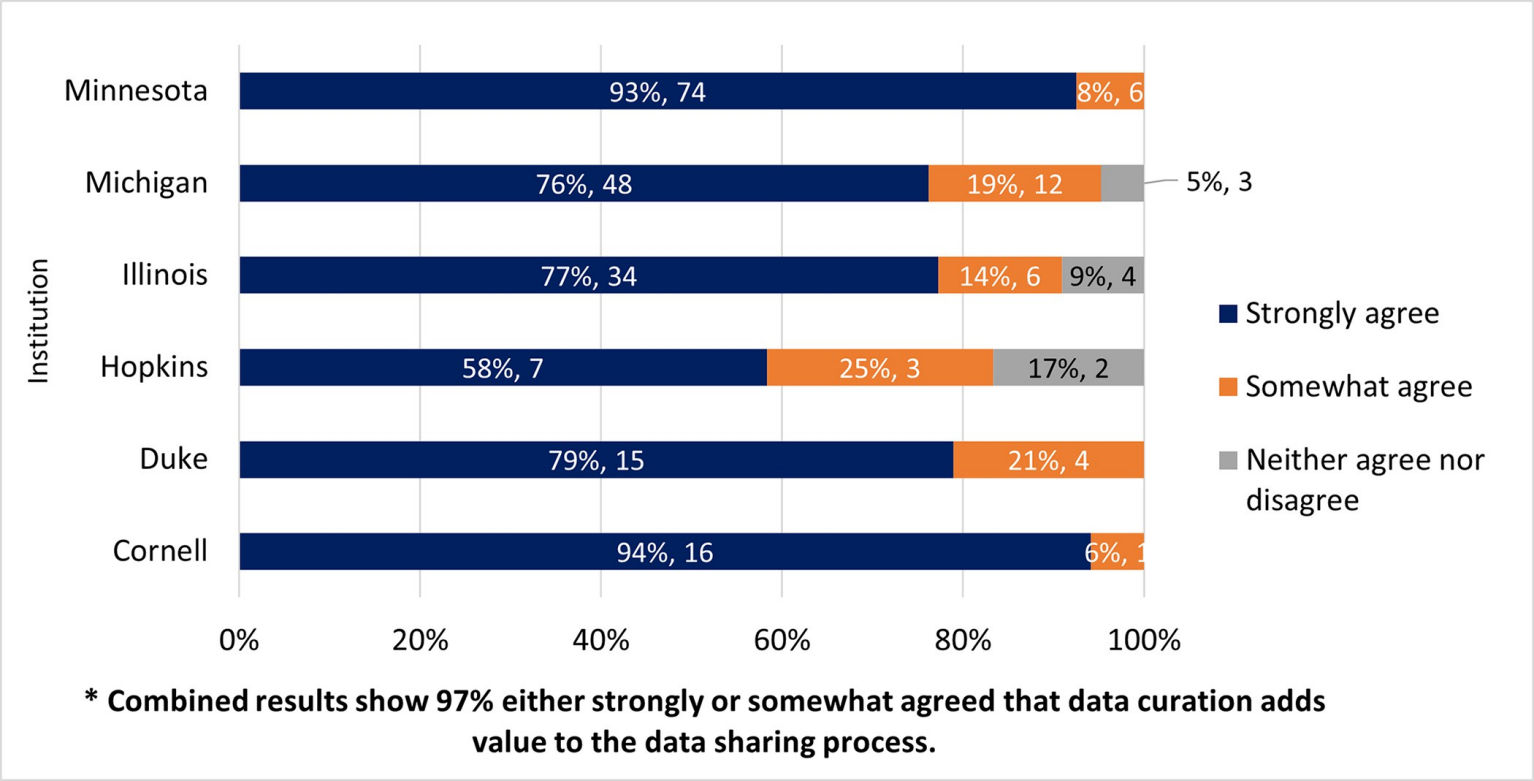
Data curation by the repository adds value to the data sharing process?. Strongly or somewhat agree; neither agree nor disagree; and strongly or somewhat disagree (zero respondents selected). The graph is displayed based on institutional responses.

Participants were asked if they agreed with the statement that data curation provided by their local repository added value to the data sharing process. There were 235 responses for this question and, 97% (n = 226) of those responding strongly (83%, n = 194) or somewhat (14%, n = 32) agreed that data curation adds value to the data sharing process, while a small fraction 4% (n = 9) were neutral (neither agreed nor disagreed), and no participants strongly or somewhat disagreed. Fig 6 displays the breakdown based on each institution’s responses.

### What is the most "value-add" curation action taken by this repository? Tell us why you think so

This question, answered by 179 participants, gave survey participants an opportunity to provide free text responses, and provided a substantial amount of qualitative data, which we summarize below. Although the question asked for specific actions adding value, 46 responses focused more generally on the benefits of curator review without identifying a specific curation action.

**Fig 7:**
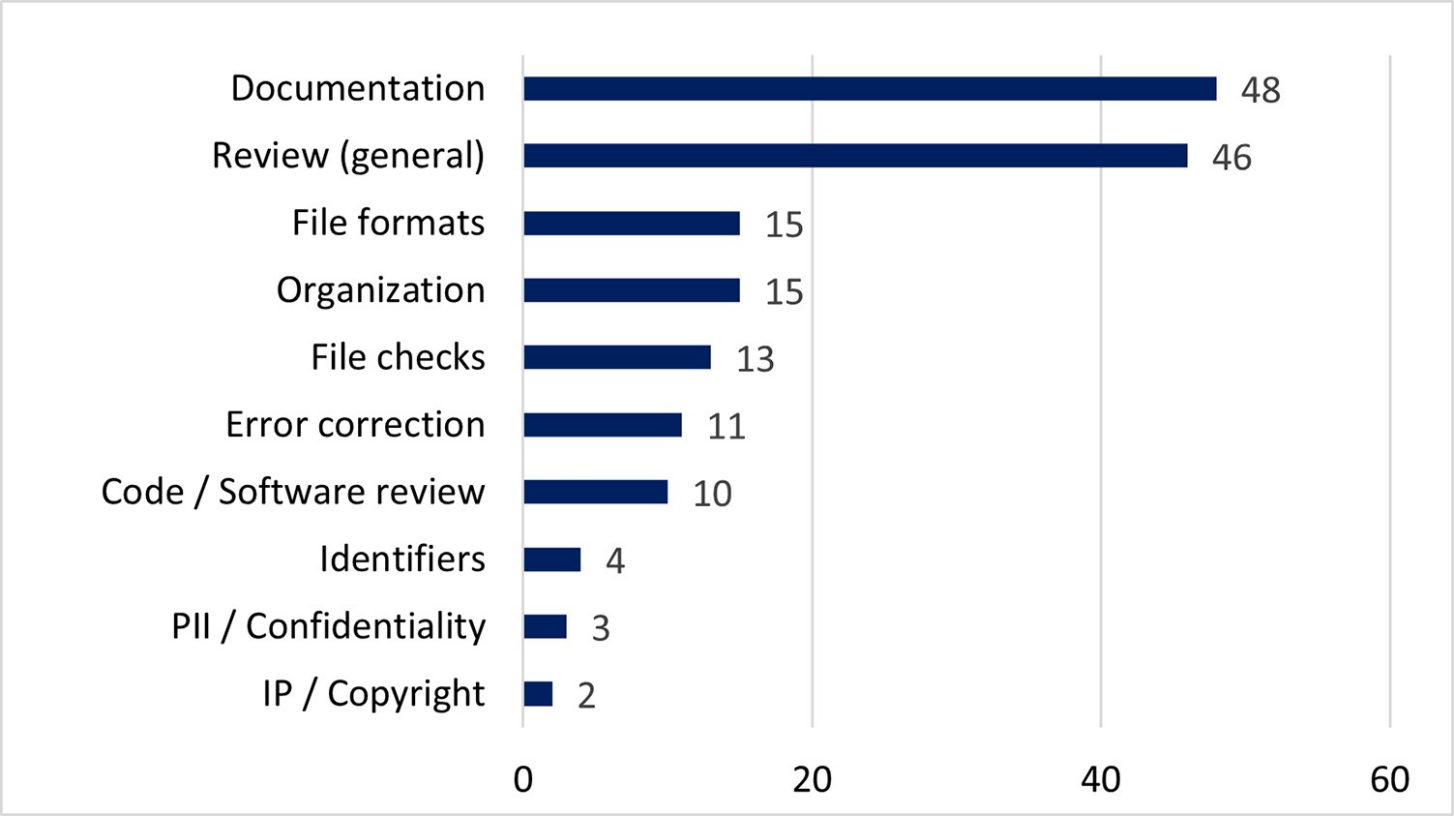
What is the most "value-add" curation action taken by this repository?. (n = 179) Free text responses coded for **curation actions**; some responses mentioned more than one curation action.

Fig 7 shows all of the curation actions mentioned by participants. The actions most frequently mentioned were creation and enhancement of documentation and metadata. It is worth noting that in some cases the use of the term “metadata” was not always clear whether the participant was referencing formal structured metadata or metadata as a general term for documentation/description; regardless, enhancement of the description of their datasets either through structured or unstructured means (i.e., readme files) was the highest valued action.

A number of actions concerned with providing file or data level checks to optimize organization, formats, identify internal errors, or review code were also mentioned, although less frequently than documentation and metadata. Finally, a set of actions that are more specific in their focus and do not apply to all datasets, e.g. checking for Personally Identifiable Information (PII) or Intellectual Property (IP) concerns, were mentioned by a few participants.

**Fig 8:**
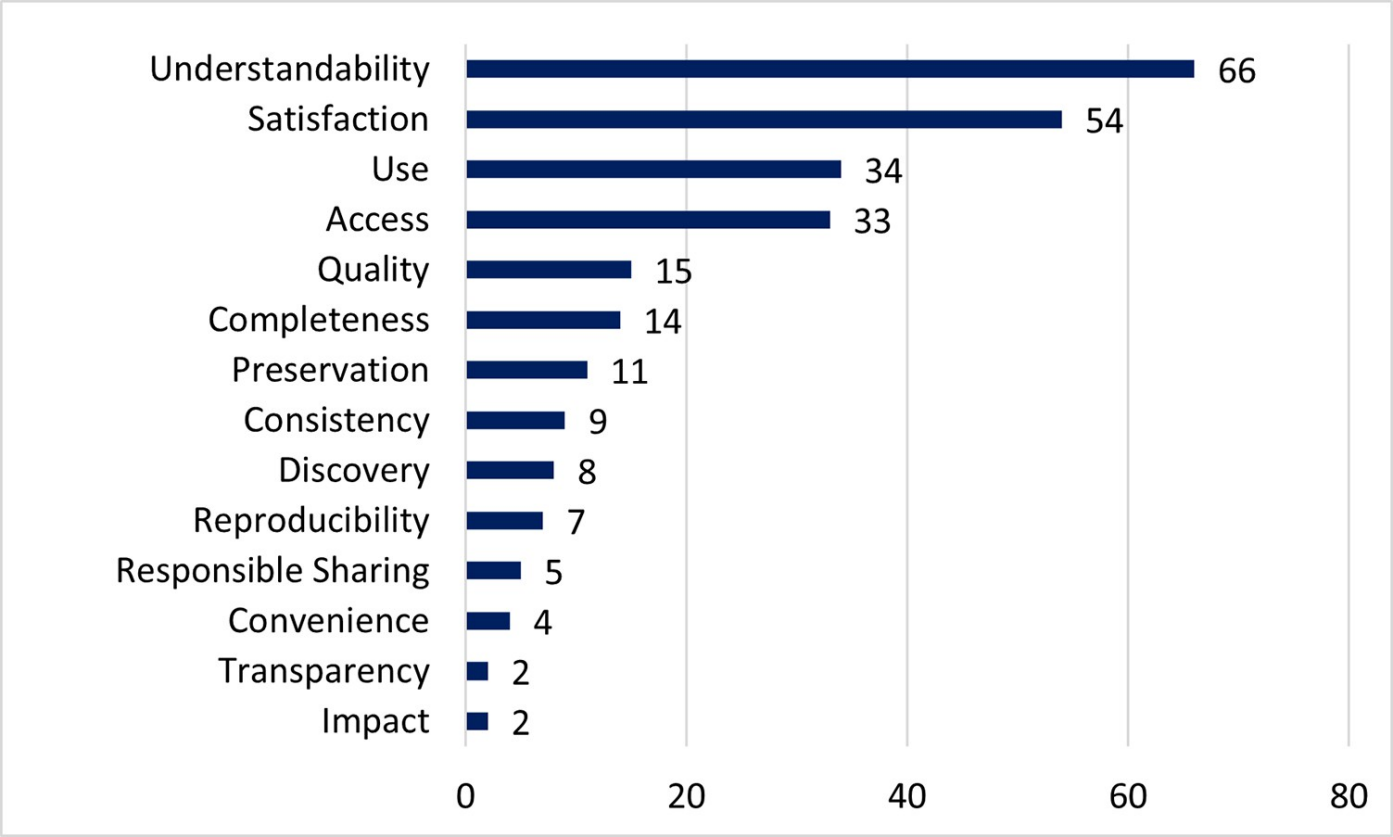
What is the most "value-add" curation action taken by this repository?. (n = 179) Free text responses coded for outcomes of curation; responses may mention more than one outcome.

Many of the free text responses also spoke to the outcomes of the curation work, shown in Fig 8. Outcomes were mentioned a total of 264 times, with 140 out of 179 free text responses including at least one outcome. Participants overwhelmingly reported that their data was made more understandable to others due to the work of data curators (66 responses). Improving the clarity of the data was a notable sub-theme of understandability expressed in the free text responses. Several researchers expressed appreciation for having someone outside the project or discipline point out jargon or other barriers to accessing, understanding, or using their datasets. As one participant put it:

“I submitted my work from the (admittedly) narrow perspective of theoretical modeling. However, the data curation specialist did a terrific job helping me "de-jargonize" my submission so that the material was accessible to broader audiences. [She] helped me organize the material in a way that is clean and clear to the audience… with her help, any scientist can now access, understand, use, and build upon my submission, which is to say, invaluable.”

This quote is one example of how the work of the data curator, as an informed generalist who thinks about how others would encounter the dataset, adds value to the dataset. Although adding and improving metadata, documentation, and other descriptive elements of the dataset were most often mentioned, other responses indicated that actions taken by curators to format data, organize files, or identify errors in the data itself, also improved the understandability of the data. Satisfaction with their experience was the second most common outcome found in the free-text responses (54 responses). Responses discussing satisfaction included details such as the thoroughness of the curator’s review, researcher confidence in their curated data, and trust in the curator.

Comments concerning improvements made to access and use of the data as an outcome of data curation were also prevalent in responses to the survey. In many of their comments, participants indicated a lack of awareness or understanding about how others would be able to engage with their dataset. In part this is due to researchers not being familiar with best practices of data sharing, as stated in their comments. But participants also mentioned not previously recognizing their data as a publishable work that would be accessed and used by others. Comments such as the following recognize the value and expertise of data curators as people who work to improve the accessibility and potential usability of the data for others:

“The data curation was helpful in that it forced me to consider the viewpoint of the end user of the data. The curators nudged me to add pieces that would help third parties navigate the data (e.g. readme file)… In retrospect this is a good thing; at the time I was focused on getting my thesis chapter out and published, and didn’t pay attention to these aspects as much as I needed to.”

Other outcomes that were mentioned by multiple participants were improvements to the completeness and quality of the data, further reinforcing the value of data curation work as a part of the publication process. In some cases, participants noted that the data they had initially submitted was missing important details, or even entire files, that would have compromised the utility of the dataset. Similarly, participants who mentioned quality noted that the curation process was invaluable for catching mistakes in the data or checking to see that the code ran as expected, as described in the following comment:

“Meticulous review of our datasets and code enabled us to catch several errors that would have hindered replication efforts by other scholars. Gaps in documentation/codebooks, which are hard for us to detect because the data is so familiar to our team, were also detected and corrected. Feedback from someone who comes to the data/documents with fresh eyes is simply invaluable.”

Evidence of meaningful engagement with curators was found in 33 (~19%) of the responses to question 9, with the curator expertise identified (15 responses) as a valuable aspect of the curation process. In four cases, researchers gave positive responses that mentioned the curator by name. Participants also mentioned limitations in their own ability to prepare their data for sharing and archiving in the repository. More than 10% of text responses (22) noted a limitation that the curation process addressed. The most commonly identified limitation was closeness to their own data. A characteristic response stated:

“…we’re so intimately familiar with it that it’s a bit challenging to think about what someone unfamiliar with the data would need to know to use the data.”

Other limitations expressed included lack of expertise in various aspects of curation, limited time or attention for the data publication process, and technical capacity. Other themes found in the free-text responses included the value of the repository itself, standards and best practices (for example “…knowledge on best archiving and data sharing practices is extremely valuable”), and providing links between the dataset and papers.

### The impact that data curation has on the data sharing process is worth the effort

**Fig 9:**
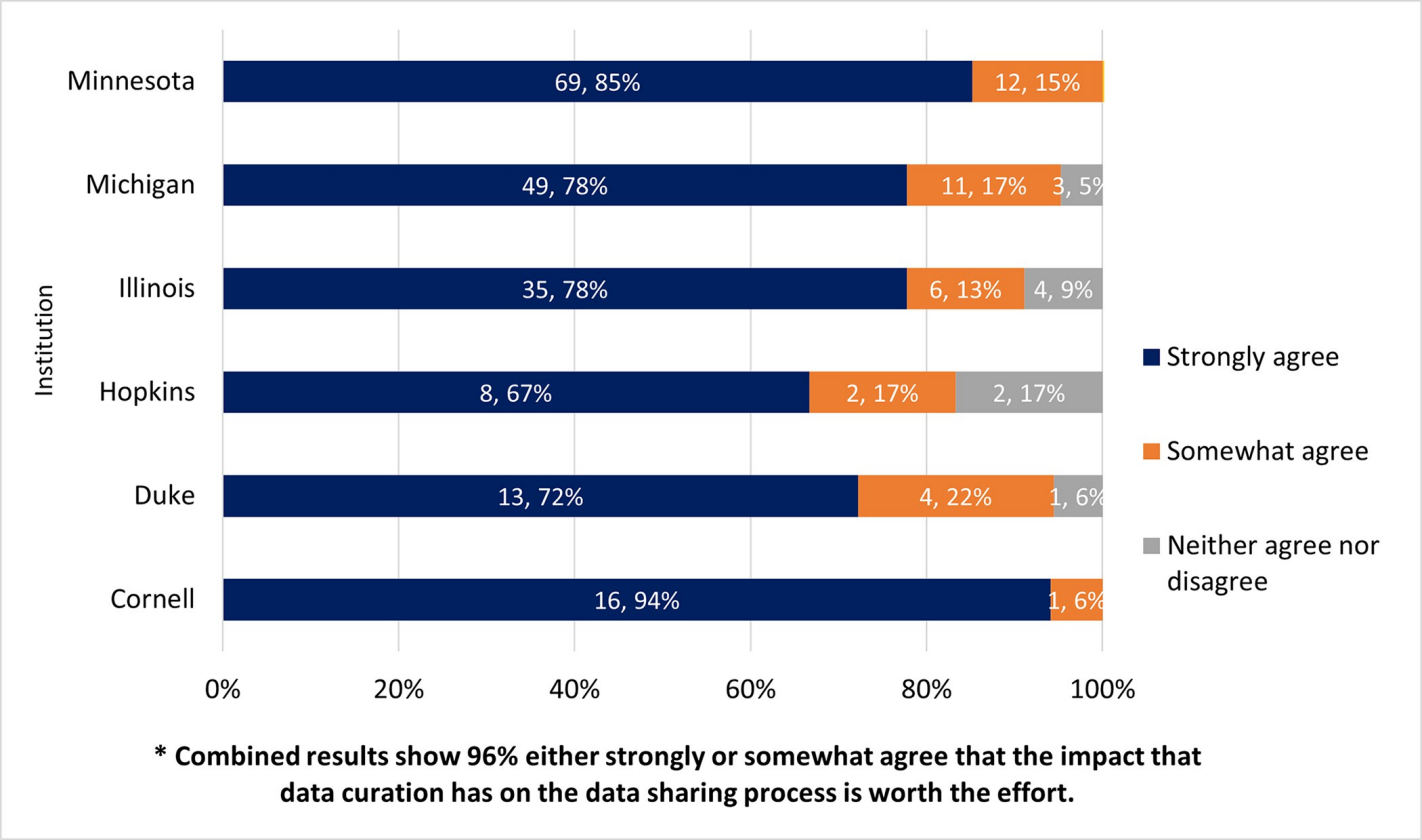
The impact that data curation has on the data sharing process is worth the effort?. Strongly or somewhat agree; neither agree nor disagree; and strongly or somewhat disagree (zero respondents selected). Graph is displayed based on institutional responses.

In question 10, participants were then asked if they agreed that the impact of data curation has on the data sharing process is worth the effort. There were 236 answers for this question with 96% (n = 226) strongly (81%, n = 190) or somewhat (15%, n = 36) agreed on this question, while 4% (n = 10) stayed neutral (neither agree nor disagree), and no participants strongly or somewhat disagreed. Fig 9 displays the breakdown based on each institution’s responses.

### Is there anything else you’d like to tell us about your data sharing experience?

The final question asked participants if there was anything else they would like to tell us about their data sharing experience. Again, many free text responses were received (n = 182). Many expressed positive satisfaction with the curation service and emphasized the value added to the data sharing process:

“I see this curation process as the "capstone" of our research, and it is invaluable for making materials readily available to future researchers. Further, the clarity of the submissions that [curator name] and her team have helped us produce will propel science in that future researchers can assess quite rapidly the applicability of our tool to their question at hand. …. I am thankful and excited for the help in curation and archival of all our research products because I see that teamwork in this final step of research means that the best possible version of the material will be available to future generations.”

And again, catching mistakes was mentioned as a benefit of curation, for example, this participant who, after curation, is more comfortable sharing their data and proud of the result:

“I try HARD to make my data good before it ever gets to the repository and every single time there’s been curation, there’s always been something that I’ve missed. I’m very grateful for such careful and helpful curation. It definitely increases my comfort and even pride in the dataset.”

Along with compliments, participants also provided suggestions to improve the local workflow, such as: consider optimizing the user experience; add repository features or improve organization; and expand communications and outreach to raise local awareness of the data repository and curation services. Some participants also raised concerns, such as the benefits of data sharing in reality; barriers of sharing due to privacy/security agreements; and how to handle situations where the data may not be a perfect fit for the repository, as the following response indicates:

“Because of privacy agreements, I was only able to share statistical code with sample data and not real data. [repository name] wasn’t a perfect match for this, [in] terms of both public messaging and the data-centric submission form. Though it was better than, say, my Github account which is much less permanent. I would value an archive which was better designed to store code in addition to data.”

### Would you recommend your colleagues submit data to this repository?

When asked if they would recommend their local repository, 98% (n = 234) responded yes.

## Discussion

This survey aimed to evaluate the satisfaction level of researchers who had received data curation services, but the authors also hoped to learn a few more things from researchers who had received curation services. Do they expect data curation? Do they make changes to the data when it’s recommended? What changes are most common? Do they feel more confident sharing their data after curation? Do they feel that data curation adds value? What curation actions are most valuable? Do they feel that data curation is worth the effort? Overwhelmingly, the answers were positive in their evaluation of the importance and value of curation, indicating that researchers not only value curation services, but are largely satisfied with them.

### Do they expect data curation?

Surprisingly, one third of respondents did not expect their data to be curated by the institutional repository. However, almost 90% strongly agreed that they were satisfied with the curation services received, with no respondents expressing dissatisfaction. This indicates that although many participants didn’t expect it, most were happy to have their data curated. As some participants indicated in their feedback, there is work to do when it comes to marketing services at the respective institutions and raising awareness of curation services. Some institutions have been wary to advertise broadly due to concerns about whether an increase in demand for curation can be handled by existing staffing levels. Can the quality of curation services stay consistently high when curators are faced with increasing numbers of datasets? As services grow, it will be necessary to continue to check in with researchers to make sure they receive high quality curation actions of value to them.

### Do they make changes to the data when it’s recommended?

At least 50% of the time, and up to 82% of the time, depending on the institution, survey participants reported that changes were made to their data due to curatorial recommendations. This finding stands in contrast to an internal assessment by Luong and her team at the University of Illinois, Urbana-Champaign of their institutional data repository, the Illinois Data bank. They found that 22% of researchers do not respond to curators [5]. We attempted to learn more about this disconnect in our survey by asking participants if any actions had been taken to make changes to their dataset as the result of curation. While around a quarter of survey participants either did not make changes or were unsure whether changes had been made, those participants that didn’t make changes overwhelmingly (94%) said that it was because no changes were needed. No participants indicated that they didn’t make changes because they disagreed with the recommendations; only one indicated that they were too busy. It may be the case that depositors who disagreed with recommendations or were too busy to make changes were also too busy to respond to this survey.

### What changes are most common?

The authors had hypothesized that when the data curation process results in a major change (e.g., curator finds a missing element in the data), then researchers are more satisfied. The results of the survey overwhelmingly indicated satisfaction with the curation experience that the authors couldn’t identify any correlation between satisfaction and the degree of changes made to the data. Interestingly, if we compare survey participants’ evaluation of the degree of the changes made with internal documentation from DCN curators collected over the same time period, curators evaluated the degree of changes made somewhat differently. Only 8% of participants indicated that essential changes (e.g., an error was corrected) were made; 27% indicated major changes (e.g., files updated/added), and the majority, or 63% indicated only minimal changes (e.g., small edits/additions) were made (n = 180). In contrast, the internal documentation collected by DCN curators over the same time period indicated that the majority of datasets submitted for network curation required major changes. In fact, for datasets curated by DCN curators between January of 2019 and June 2021, curators indicated that of datasets requiring changes (n = 210), 55% required major changes, 40% required minimal changes, and 6% required essential changes (internal ticketing data provided by DCN coordinator). There is substantial overlap between the datasets in question, and both groups were using the same definitions for the changes made, however the assessment of the extent of changes made is coming from two different points of view: that of the researcher/depositor/survey participants and that of the DCN curators, so it is interesting that survey participants considered the changes made less serious than curators did. For the two groups, a very similar percentage of the datasets did NOT require changes, with 25% (59) of survey participants responding no or unsure to changes being made (Fig 3), and 23% (49) of curator-evaluated datasets not requiring changes. This is admittedly a rough comparison, and would benefit from real one-to-one comparison of the same datasets as assessed by the submitter and the curator. Future research could dig into differences in how curators and researchers evaluate changes made to data and how that affects satisfaction, both for researchers and for curators.

### Do they feel more confident sharing their data after curation?

The authors also hypothesized that researchers would be more confident sharing their data after curation, and that they would feel that data curation adds value. Overwhelmingly participants agreed with both of these statements in the survey, and provided comments to back that sentiment up:

“I felt so much more confident knowing that a data curation expert was reviewing my deposit…,”

“Though no changes were made to this dataset, that’s only because I’d worked with the team on previous data deposits. Having access to knowledge on best archiving and data sharing practices is extremely valuable.”

Unexpectedly, personal engagement came up in survey responses multiple times. Four participants mentioned their curator by name, indicating an appreciation for personalized engagement and the human labor involved in curating datasets. Even when no names were mentioned, many participants mentioned the importance of a curator specifically, such as this comment:

“It’s good to have someone who is not intimately familiar with the research give a "reality check" on whether or not enough details are provided in the metadata to facilitate use of the deposited datasets by other researchers.”

### Do they feel that data curation adds value?

The majority (97%) of survey participants agreed either strongly or somewhat that data curation adds value, clearly indicating the importance of curation to researchers. Furthermore, more than 75% of the survey participants expressed satisfaction with the curation services being provided. This is very encouraging when compared to the focus groups held in 2018 when only 18% of participants indicated they were satisfied with data curation services overall [3]. A lot has changed since 2018; many of the institutions involved have worked to establish and or improve data curation services during this time. For example, Cornell University had no formal curation service before 2018. The Data Curation Network project, which was launched across the six pilot institutions in 2018, intended to address the need for sustainable curation services across the participating institutions. While there are large differences in the way this survey was delivered, the number of participants, and even the questions asked, it is nonetheless encouraging to note that in 2021, three quarters of survey participants expressed satisfaction with data curation services.

### What curation actions are most valuable?

The focus groups reported in 2018 identified 12 data curation activities that were most important to researchers, and comments from participants expressed frustration and a “desire for greater support in many data curation activities” [3]. The results of this survey revealed positive updates—both with respect to which data curation activities were most important to researchers and how satisfied they were with data curation services in general. In this survey, documentation was most frequently mentioned as the most “value add” curation action (48 responses, 27%). Documentation was also important to researchers in the 2018 focus group study, but only slightly more than a quarter said “yes” that they were satisfied with the documentation of their data; while nearly half of them were “somewhat” satisfied (possible answers were yes, no, or somewhat) [3]. Metadata fared similarly, in the current survey, 46 responses (26%) named metadata as the most “value add” curation action. Five years ago, metadata was viewed as important, but with only 29% participants saying “yes” they were satisfied [3]. We acknowledge that the methods, goals, and targets are different between the two studies, yet it is still encouraging to see such a large difference in satisfaction expressed by the researchers five years later. The Data Curation Network was created to better meet researcher needs around data curation, and the survey responses indicate that researchers who have utilized our curation services recognize the importance of documentation, metadata, and other curation activities and furthermore are satisfied with the results. Education and awareness may also be playing a role—the move towards open science and increasing requirements around data sharing by funders and publishers could be making researchers more aware of the need for curation and more appreciative of our services as well as the end result.

### Do they feel data curation is worth the effort?

The answers to the survey provide a resounding “yes” to a degree that surprised us. Almost no participants disagreed, the majority strongly agreed that data curation is worth the effort. This researcher sums it up nicely:

“[I w]as initially reluctant about some open data impulses in my field as just another step in the publishing process that wasn’t likely to be used or useful in most cases. But the [repository name] has been so easy to use and the feedback on datasets so helpful that it’s made me feel much more confident just publishing datasets before we even go to journal submission and linking DOI with my manuscripts. It’s also addressed some of my skepticism that anyone would care; one of the first datasets we provided to the [repository name] was downloaded >400 times last I checked. Which was very exciting; it was a data product we hoped other people really would use in that case, but it was rewarding to the graduate student to see that GIS files she had developed to help land managers in our study region really were being downloaded. It was neat.”

## Study limitations and future research

While the authors are gratified to report the positive responses received to this survey, it is important to note that this is a survey of only researchers who deposited data in six specific repositories over a specific time period. This leaves out a wide swath of researchers who may have very different opinions and awareness of the value of curation. Furthermore, although the response rate was high, it is possible that the researchers who were motivated to respond to the survey were those who benefited most from curation services. Especially when considering that only one response mentioned time limitations as a reason for not making changes to the data, it was hypothesized that many of the researchers who did not make recommended changes to their data due to time limitations or other considerations also did not respond to the survey for those same reasons. Other method(s) may need to be utilized to get feedback from researchers who are unaware of the services, too busy to take advantage of the services, or feel that only they are qualified to curate their own data.

Our survey respondents include a mixture of researchers receiving data curation from curators affiliated with their home repository and DCN curators affiliated with other institutions. Although we did not find evidence to suggest that curator affiliation affected user experience, our sample sizes and the number of repositories involved did not allow for statistical comparison between DCN and local curation. The emphasis placed on both well-defined curator workflows and communication within the Data Curation Network [1,26] is meant to provide researchers a smooth, professional experience regardless of curator affiliation. Further research is needed to evaluate whether researchers perceive a difference when the curator is not affiliated with the home institution. Additional questions for future research include: Why do researchers choose to share their data in the local institutional repository? How can workflows be improved for shared curation services? Are there other data-related services that could be improved or made more sustainable using a collaborative approach? How can curators engage and learn from researchers who aren’t currently taking advantage of curation services?

## Conclusion

The survey response rate was high, and the responses themselves indicate satisfaction among end-users of the institutional repositories and respective curation services. At the outset of the Data Curation Network project, focus groups indicated that researchers were aware of and actively engaged in a variety of data curation activities, but few to none of those data curation activities were happening in a satisfactory way for the majority of the researchers [3]. While this survey approaches the question using a different method, and there may still be researchers for whom data curation services are not satisfactory, these results indicate that data curation services associated with institutional data repositories have had a positive impact on the researchers surveyed. The Data Curation Network was created to better meet researcher needs around data curation, and the survey responses indicate that researchers that have utilized our curation services are satisfied.

While curation is an unexpected service for some researchers who deposit data in our institutional repositories, the majority of researchers expressed satisfaction with the service. Furthermore, researchers described multiple challenges that curators assist with in the process of sharing their data, and identified, in their own words, valuable outcomes of working with curators such as increased understandability, use, and access.

This survey and its results are being shared because it offers an example for others in the repository community wishing to obtain depositor feedback on their local curation process. Furthermore, these insights should be of interest to researchers, publishers, funders, and institutions interested in exploring and increasing the return on investment of data sharing. The detailed responses illustrate the many ways that curation helps to reduce the burden on researchers and increase the likelihood that data is not just shared, but is made more findable, accessible, interoperable, and reusable. These results may also be helpful for communicating the value of curation services externally to researchers and internally to administration, institutional leadership, and beyond: **97% of researchers agreed that data curation adds value to the data sharing process, 96% agreed it was worth the effort, and 90% felt more confident sharing their data due to the curation process.** This, along with the detailed responses may help to identify gaps and opportunities–for funders and institutions developing new data sharing policies, repository managers considering whether adding curation workflows is worth the effort, curators marketing existing services, or those who are fine-tuning or evaluating curation services.

## Supporting information

S1 Appendix Qualitative analysis free-text coding theme definitions. (PDF)
