# Supplementary material for: Understanding the value of curation: A survey of researcher perspectives of data curation services from six US institutions
Source: PLoS One. 2023 Nov 1;18(11):e0293534. doi: 10.1371/journal.pone.0293534 (PMC10619857; doi:10.1371/journal.pone.0293534)
Supplement: S1 Appendix — (PDF) [file pone.0293534.s001.pdf]

S1 Appendix. Qualitative analysis free-text coding theme definitions.

Use	Definition
Any theme followed with "Not done"	Response mentions this theme, but specifically mentions that the action is NOT taken. May be important to the repo, but for various reasons is not happening, or does not apply to their situation, though might in the future.
3rd party reviewer	Response makes a general mention of having a 3rd party review their data
3rd party reviewer | New set of eyes	Response refers to the benefit of having another person (set of eyes) review and provide feedback on the data set
3rd party reviewer | Outside of field	Response refers to the benefit of having another person (set of eyes) from outside of the research field or discipline of the researcher review and provide feedback on the data set
Curation action | Code runs / Software	Response indicates a action that involves ensuring that code runs or that software/code are reviewed
Curation action | Documentation	Response refers to reviewing, checking, or verifying documentation, including readme files.
Curation action | Error correction	Response indicates mistakes were corrected (if response specifies what was corrected, can also add themes to represent those)
Curation action | File formats	Response indicates an action that involves recommendations or modifications to file formats
Curation action | File checks	Response indicates a action that involves examination of data file contents themselves (e.g file format validation, virus checks, addressing missing values/codes)
Curation action | Metadata	Response refers to reviewing, checking, or verifying metadata content and/or links.
Curation action | Metadata | identifiers	Response indicates a action that involves metadata improvement by addition of a persistent identifier , such as a DOI
Curation action | Organization	Response indicates an action that involves data or file organization, including structure, file naming, etc.
Curation action | PII / Confidentiality	Response indicates an action that involves handling human subject or other sensitive types of data
Curation action | Review (general)	Response describes review in general without specifying what (metadata, documentation or data files) exactly was reviewed
Curation action | Specialized Services	Response describes a specific, specialized curation action (e.g. Globus, etc) that is not defined in our other coding buckets

Data & Papers	An indication that a data set has some form of connection to an article, a RIMS or similar output (eg. links).
Engagement | Curator	An indication that the depositor interacted with the curator as a person, in some meaningful way.
Engagement | Curator | Curator Guidance / Education	A reference to the education delivered to the depositor by the curator they were working with or that learning occurs via the process
Engagement | Curator | Expertise	A reference to the expertise or perspective of the curator doing the review
Engagement | Curator | Named	Response directly names a curator
Engagement | Peer review	An indication that the curator or repository enables interaction with a peer-reviewer.
Goal (Outcome) | Access	An outcome of the curation work is to provide access to the data. Also, used when data sharing is mentioned by the researcher in the comment.
Goal (Outcome) | Completeness	An outcome of the curation work is to ensure data is "complete" or has all of the necessary components to be shared with others; this could include any necessary software used for processing
Goal (Outcome) | Convenience	An outcome of the curation work is that it makes life easier for the researcher in some way
Goal (Outcome) | Consistency	An outcome of the curation work is that the data set is consistent throughout the documentation and/or between files
Goal (Outcome) | Impact	An outcome of the curation work is that the data set will further the impact of the research or the researcher
Goal (Outcome) | Preservation	An outcome of the curation work is to ensure the longevity of the data
Goal (Outcome) | Quality	An outcome of the review is that data and or metadata meets a specified level of quality or integrity according to the researcher in their response.
Goal | Reproducibility	A goal of the curation work of the repository is reproducibility.
Goal | Responsible Sharing	An outcome of the curation work is to ensure data is shared responsibly.
Goal (Outcome) | Satisfaction	An outcome of the curation work is satisfaction (mentioned directly or strongly indicated in the response itself)
Goal (Outcome) | Satisfaction | Confidence	An outcome of the curation work that leads to more confidence in sharing the data generally or having others make use of it successfully (mentioned directly or strongly indicated in the response itself)
Goal (Outcome) |	An outcome of the curation work is satisfaction due to the thoroughness of

Satisfaction | Thoroughness	the curation review (mentioned directly or strongly indicated in the response itself)
Goal (Outcome) | Satisfaction | Trust	An indication that the researcher trusts the expertise and actions taken by the curator to improve or add value to their data set
Outcome | Transparency	An outcome of the curation work is more transparency in the research process
Goal (Outcome) | Understandability	An outcome of the curation review is that others would be able to understand or comprehend the data set
Goal (Outcome) | Understandability | Clarity	An outcome of the curation work is to ensure data is "clear" or has sufficient information or context to be understood by others.
Goal (Outcome) | Use	An outcome of the curation work of the repository is use (or re-use).
Goal (Outcome) | Use | cross-disciplinary	An outcome of the curation work of the repository is cross-disciplinary use (or use outside of the original discipline).
Limitation | Closeness to the Data	Response includes a statement on the difficulty of researchers editing and curating their own data due to their having worked with it for so long and/or so in depth
Limitation | Expertise	Response indicates a limitation due to skills or expertise of the depositor
Limitation | Technical capacity	Response indicates a limitation due to some technical capacity (ex. running the code in just their environment).
Limitation | Time or Attention	Response indicates a limitation due to a lack of time to work on the data, or attention paid to data sharing preparation or the data itself
Repository	Response mentions a repository infrastructure or its services in general terms
Standards / Best Practices	Response refers to a specific standard or best practice for their data
